# Morning light treatment for inflammatory bowel disease: a clinical trial

**DOI:** 10.1186/s12876-024-03263-2

**Published:** 2024-05-22

**Authors:** Shirley Cohen-Mekelburg, Cathy A. Goldstein, Muneer Rizvydeen, Zainab Fayyaz, Priya J. Patel, Jeffrey A. Berinstein, Shrinivas Bishu, Kelly C. Cushing-Damm, Hyungjin Myra Kim, Helen J. Burgess

**Affiliations:** 1 Division of Gastroenterology and Hepatology, University of Michigan Medicine, 1500 East Medical Center Drive, Ann Arbor, MI 48109 USA; 2 VA Center for Clinical Management Research, VA Ann Arbor Healthcare System, Ann Arbor, MI USA; 3 Institute for Healthcare Policy and Innovation, University of Michigan, Ann Arbor, MI USA; 4 Department of Neurology, University of Michigan, Ann Arbor, MI USA; 5 Sleep and Circadian Research Laboratory, Department of Psychiatry, University of Michigan, Ann Arbor, MI USA; 6 Consulting for Statistics, Computing and Analytics Research, University of Michigan, Ann Arbor, MI USA; 7 Department of Biostatistics, University of Michigan, Ann Arbor, MI USA

**Keywords:** Crohn’s disease, Ulcerative colitis, Sleep, Light treatment

## Abstract

**Background:**

Inflammatory bowel disease (IBD) affects over 3 million Americans and has a relapsing and remitting course with up to 30% of patients experiencing exacerbations each year despite the availability of immune targeted therapies. An urgent need exists to develop adjunctive treatment approaches to better manage IBD symptoms and disease activity. Circadian disruption is associated with increased disease activity and may be an important modifiable treatment target for IBD. Morning light treatment, which advances and stabilizes circadian timing, may have the potential to improve IBD symptoms and disease activity, but no studies have explored these potential therapeutic benefits in IBD. Therefore, in this study, we aim to test the effectiveness of morning light treatment for patients with IBD.

**Methods:**

We will recruit sixty-eight individuals with biopsy-proven IBD and clinical symptoms and randomize them to 4-weeks of morning light treatment or 4-weeks of treatment as usual (TAU), with equivalent study contact. Patient-reported outcomes (IBD-related quality of life, mood, sleep), clinician-rated disease severity, and a biomarker of gastrointestinal inflammation (fecal calprotectin) will be assessed before and after treatment. Our primary objective will be to test the effect of morning light treatment versus TAU on IBD-related quality of life and our secondary objectives will be to test the effects on clinician-rated disease activity, depression, and sleep quality. We will also explore the effect of morning light treatment versus TAU on a biomarker of gastrointestinal inflammation (fecal calprotectin), and the potential moderating effects of steroid use, restless leg syndrome, and biological sex.

**Discussion:**

Morning light treatment may be an acceptable, feasible, and effective adjunctive treatment for individuals with active IBD suffering from impaired health-related quality of life.

**Trial registration:**

The study protocol was registered on ClinicalTrials.gov as NCT06094608 on October 23, 2023, before recruitment began on February 1, 2024.

## Background

Inflammatory bowel disease (IBD) is a chronic immune-mediated condition of the gastrointestinal tract that includes two subtypes, ulcerative colitis (UC) and Crohn’s disease (CD). IBD affects over 3 million Americans and IBD-related healthcare use is rising with an estimated cost to the U.S. health system of over $25.4 billion annually [1, 2]. Patients with IBD suffer from relapsing and remitting flares with gastrointestinal symptoms such as diarrhea, rectal bleeding, and abdominal pain as well as extraintestinal symptoms such as sleep and mood disturbances [3]. In fact, an estimated 56% of IBD patients report poor sleep quality [4] and mood disorders such as depression affects at least 20–30% of patients with IBD [5–8]. 

Immune targeted medications such as biologics are effective at controlling IBD symptoms, reducing the likelihood of IBD flares, and preventing IBD-related complications such as surgery [9–11]. However, despite the availability of effective medical treatments, patients with IBD continue to suffer from high rates of suboptimal disease control and symptomatic flares, and persistent extraintestinal manifestations such as fatigue, depression, poor sleep, and reduced health-related quality of life [12–17]. Thus, novel adjunctive treatments are needed to better manage gastrointestinal and extraintestinal symptoms of IBD and improve health-related quality of life for patients with IBD.

There is evidence that circadian disruption (disruption of the “body clock”) is present in IBD and is associated with worse disease activity and symptoms. A summary of the literature examining the influence of circadian factors on IBD-related outcomes in tissue, rodent, and human models is summarized in Table 1. This body of research suggests that circadian disruption may be an important modifiable treatment target in patients with IBD. As light is the strongest environmental signal affecting circadian timing [18], morning light treatment in combination with a regularly timed sleep schedule is a key approach to reducing circadian disruption in humans. Morning light treatment can phase advance (shift earlier) circadian timing, and when administered around habitual wake time will usually phase advance circadian timing by ~ 1 h [19, 20]. In addition to reducing circadian disruption, meta-analyses have confirmed that morning light treatment improves mood (reduces nonseasonal depression) with medium effect sizes similar to those observed with pharmacological antidepressants [21, 22]. Similarly, a meta-analysis indicates that morning light treatment improves sleep with medium effect sizes [23]. Importantly, while light treatment is associated with some side effects (headache, eyestrain, nausea, agitation [24]), these often spontaneously remit [24, 25], and patients rarely discontinue treatment due to side effects [25]. Furthermore, light devices (with UV filter) are considered safe with no changes in ophthalmologic exams observed after 6 years of daily use (in fall and winter months) [26]. Thus, morning light treatment, a treatment that can reduce circadian disruption, has potential to enhance usual treatment and improve symptoms and disease activity in IBD with minimal side effects. Furthermore, light treatment devices are commercially available and light treatment can be self-administered at home, allowing easy access and dissemination once efficacy is established.

**Table 1:** A summary of the literature exploring circadian factors in IBD-related models

Model	Reference	Circadian Factor	Summary of Findings
Tissue	Palmieri et al. 2015; [60] Weintraub et al., 2020; [61] Liu et al. 2017 [62]; Mosna et al. 2021[63]	Clock gene expression in colonic mucosa, leukocytes	Altered expression in IBD and in inflamed mucosa, associated with increased endoscopic disease activity, inflammation
Rodent	Eum et al. 2023; [64]	Circadian disruption (constant light exposure)	Increased intestinal epithelial permeability, altered expression of tight junction proteins
	Tran et al. 2021; [65] Voigt et al. 2014; [66] Liu et al. 2021; [67] Preuss et al. 2008 [68]	Circadian disruption (shifting light/dark cycles)	Increased intestinal epithelial permeability; altered gut microbiota; reduced resistance to colonic injury
	Kyoko et al. 2014; [69] Stokes et al. 2017; [70] Liu et al. 2021^67^	Clock gene mutation	Altered expression of tight junction proteins, altered resistance to colonic injury; altered intestinal regeneration
Human	Burgess et al. 2010; [71] Conley et al. 2020 [72]	Melatonin rhythms	~ 25–73% of IBD patients had disrupted melatonin rhythms
	Chakradeo et al. 2018; [73] Swanson et al. 2021 [74]	Variability in sleep timing	Observed more in IBD patients vs. controls (whether inactive or active disease), associated with more severe IBD disease history, more intestinal permeability, more pro-inflammatory gut microbiota
	Chakradeo et al. 2018; [73] Chrobak et al. 2018 [75]	Later chronotype/more eveningness	Associated with reduced IBD-related quality of life, increased fatigue

**Fig. 1:**
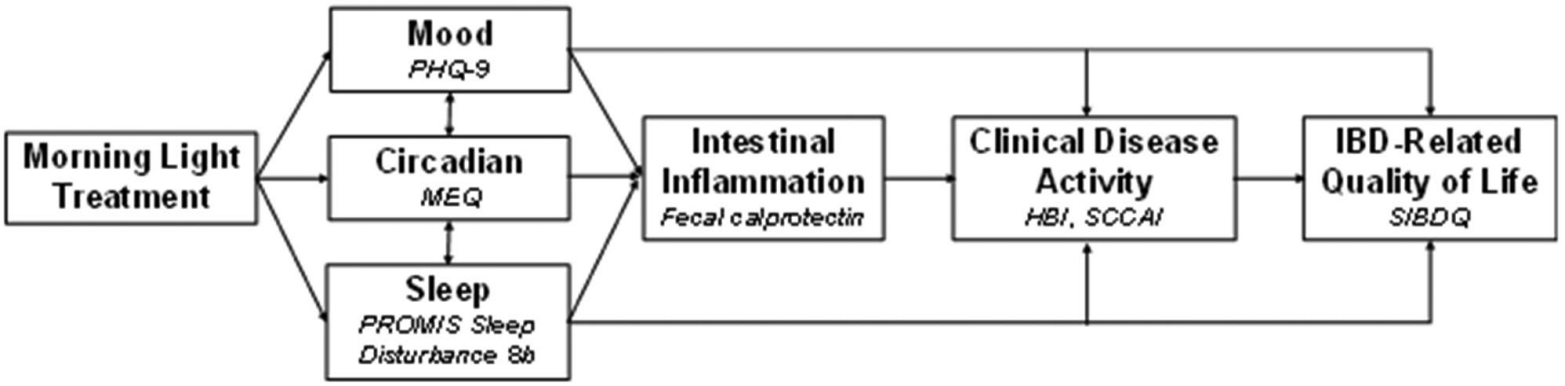
A conceptual model illustrating that morning light treatment, which is known to improve mood and sleep and to reduce circadian disruption, may also reduce intestinal inflammation and clinical disease activity, all with potential to ultimately improve IBD-related quality of life. The measure used to assess each domain is shown in italics: Patient Health Questionnaire (PHQ-9), Morningness-Eveningness Questionnaire (MEQ), PROMIS Sleep Disturbance 8b, Harvey Bradshaw Index (HBI), Simple Clinical Colitis Activity Index (SCCAI), and Short Inflammatory Bowel Disease Questionnaire (SIBDQ)

Our research group has previously tested a wearable commercially available light device (Re-timer®) to administer morning light treatment to people with chronic pain conditions and found good acceptability, feasibility, and significant improvements in mood, sleep, and pain outcomes [27, 28]. However, there are no studies testing the effects of morning light treatment on IBD disease activity, symptoms, or health-related quality of life. Given the known impact of morning light treatment on reducing circadian disruption and improving sleep and mood, we hypothesize that morning light treatment has potential to improve IBD-related quality of life directly, and potentially also clinical disease activity by reducing intestinal inflammation (see conceptual model in Fig. 1).

To test this, we will conduct a single-center randomized controlled trial for patients with IBD to test the effect of 4 weeks of morning light treatment on IBD-related quality of life (primary outcome), mood (depression), sleep, and clinical disease activity. In addition, we will explore the effect of morning light treatment on gastrointestinal inflammation and identify any possible moderating effects of sex, corticosteroid use, or restless leg syndrome (which occurs in 20–30% of IBD patients) on quality of life [29–31]. We hypothesize that participants who receive morning light treatment will report greater improvement in clinician-rated disease activity and patient reported outcomes than participants receiving treatment as usual (TAU). The study findings will provide novel preliminary data on the acceptability, feasibility, and efficacy of morning light treatment in IBD to inform a future large-scale confirmatory trial.

## Methods/Design

### Study design

**Fig. 2:**
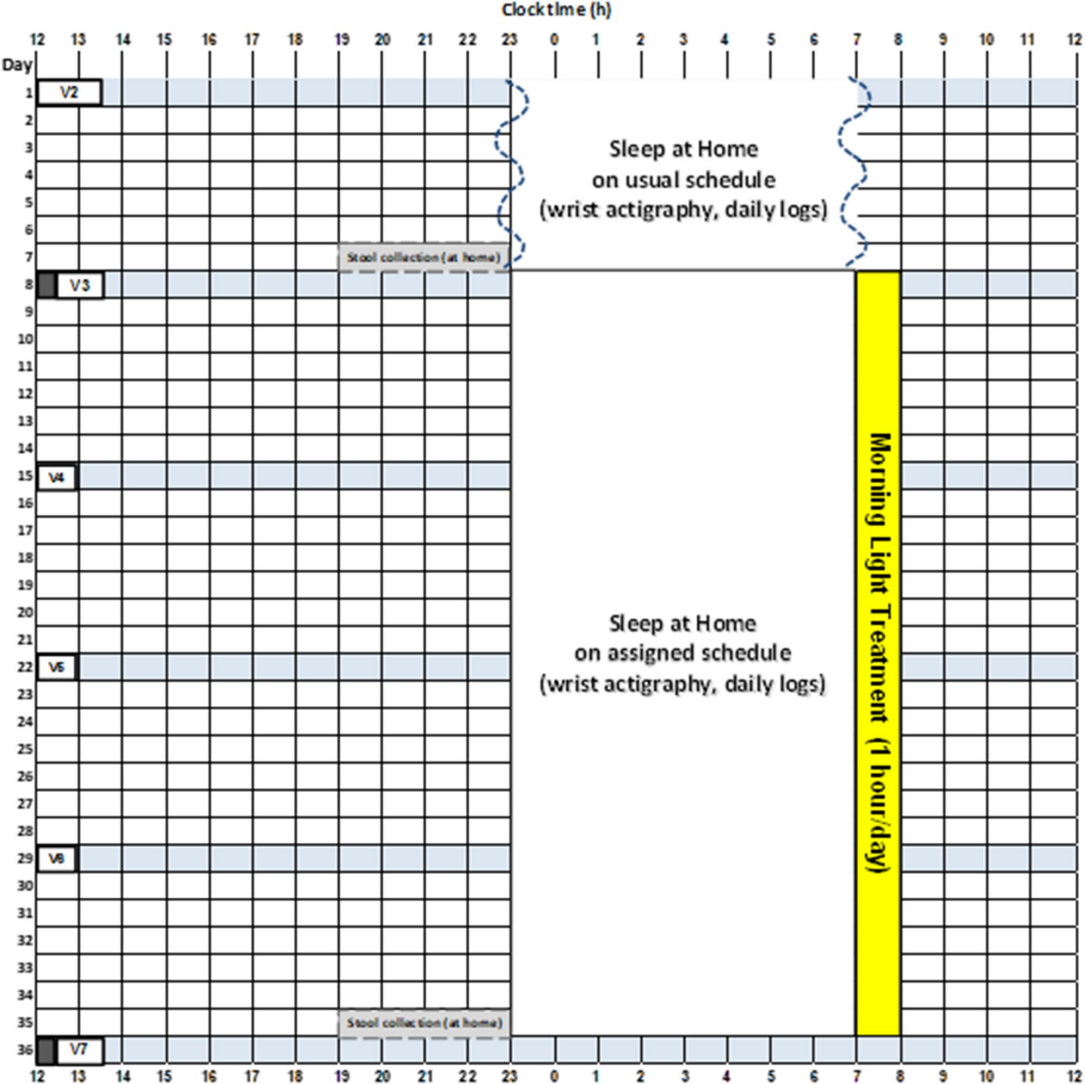
A diagram of the 5-week study protocol for a participant with an average sleep schedule of 11pm to 7am who is assigned to the morning light treatment. During visit 2 (V2), participants receive a wrist actigraphy monitor and are instructed on how to complete daily logs while sleeping ad lib at home. A week later at visit 3 (pre-treatment, V3) participants bring in a stool sample collected at home the day before, outcome measures are collected (including the clinical disease activity assessment represented by the dark rectangle), and participants are randomized to morning light treatment or treatment as usual (TAU). During weekly visits (V4, V5, V6) participants’ adherence to the light treatment and sleep schedule are checked (if assigned to morning light treatment) and daily logs and side effects are reviewed (both groups). A week later during visit 7 (post-treatment, V7) participants bring in a stool sample collected at home the day before, and post-treatment outcome measures are collected again. The TAU study protocol looks similar to this morning light treatment protocol except the ad lib sleep during the first week of the study continues throughout the 5 week study protocol. The anticipated duration of each study visit is shown

The Inflammatory Bowel Disease (IBD) Sleep Study is a prospective single-center clinical trial with a two-arm parallel groups, randomized design comparing 4 weeks of morning light treatment versus treatment as usual (TAU) in patients with IBD. The TAU group is presented to participants as a study of sleep timing to minimize group differences in treatment expectations. A sample diagram of the study protocol is shown in Fig. 2. There are weekly study visits during the 5-week protocol, with outcome variables assessed after a week of sleep monitoring at pre-treatment (visit 3) and then 4 weeks later at post-treatment (visit 7).

### Setting

Study participants will be recruited from the University of Michigan Health System. The University of Michigan Health System is comprised of hospitals, health centers, and clinics owned and operated by the University of Michigan in Southeast Michigan and conducts approximately 1.6 million ambulatory care visits annually. The IBD team at the University of Michigan comprises one of the largest IBD-specialized centers in the United States and cares for over 8,000 patients with IBD each year. With IRB approval, potentially eligible participants will be identified through a review of electronic medical records. They will be emailed a study flier, with a follow up phone call a few days later. The study is also advertised on the University of Michigan Research Participant Registry (umhealthresearch.org). All study visits occur at the Sleep and Circadian Research Laboratory in the Department of Psychiatry at the University of Michigan.

### Study population

We aim to enroll 68 individuals with a biopsy-proven diagnosis of IBD, aged 18 years or older, who meet criteria for impaired IBD-related quality of life and report recent abdominal pain or bowel symptoms (Table 2). Impaired IBD-related quality of life will be reflected by a Short IBD questionnaire (SIBDQ) score < 60. We have chosen inclusion/exclusion criteria to permit as many people with symptomatic IBD as possible to participate safely, while maximizing generalizability of findings (Table 2). For example, prescribed hypnotics, over-the-counter sleep aids, and antidepressants will be permitted as their use is common in IBD, but these permitted medications will need to be stable for 30 days before and during the study. Changes in medications to treat IBD during follow-up will be permitted as this is typical in clinical practice (although changes in corticosteroids will be cause for exclusion due to impact on sleep quality which could confound study results, see Table 2). Participants will be able to continue psychological therapy, physical therapy, and exercise if the treatment was started 30 days prior to enrollment and continues during the study period. Participants must be physically able to travel for the study visits and have no experience with light treatment in the past year. Participation will be scheduled ≥ 1 month from night work, travel outside the eastern time zone, other research participation, and will be scheduled during a period with minimal special events (e.g., weddings, concerts, exams).

**Table 2:** Inclusion and exclusion criteria

Inclusion criteria
1. Age ≥ 18 years old
2. Biopsy-proven IBD
3. Impaired quality of life: Short IBD Questionnaire (SIBDQ) < 60
4. Symptoms: at least “some of the time” abdominal pain or bowel symptoms on SIBDQ
5. Fluent in English
6. Physically able to travel to study visits, within 1.5 h drive of study location
**Exclusion criteria**
Health:
1. Current ileostomy, colostomy, ileoanal pouch, ileorectal anastomosis, or short bowel syndrome
2. Other significant chronic physical disease (e.g., uncontrolled diabetes, advanced liver disease, cancer, kidney failure, uncontrolled cardiovascular disease, seizures, light-triggered migraines).
3. Has a pacemaker or defibrillator (potential interference from Fitbit device)
4. Retinal pathology, cataracts, glaucoma, colorblindness
5. Lifetime history of psychotic or bipolar disorders
6. Suicidality in past 6 months
7. Alcohol or substance use disorder in past 3 months (cannabis use ≤ 1/week ok)
8. High risk for or diagnosed with obstructive sleep apnea and/or narcolepsy
9. Severe hearing problem, cognitive impairment, not fluent in English
10. Pregnant, trying to get pregnant, or breastfeeding
11. Has a child or pet at home that disturbs sleep often
12. Pending medical leave application at work, pending legal case/litigation
13. Working shiftwork that affects sleep
14. Recent (< 1 month) travel outside Eastern Time Zone
15. Participating in another research study
Medications:
17. Taking photosensitizing medications to blue/green light (including methotrexate, sulfasalazine, promethazine, prochlorperazine)
18. Taking melatonin
19. Unstable non-IBD medication use 30 days prior to or during study
20. Change of ≥ 10 mg/day of steroid medications use 30 days prior to or during study
Light treatment:
21. Average wake time is before 5:00am
22. Unable to fit 1 h of light treatment into morning routine
23. Previous use of light treatment device in the past year

### Procedures and assessments

Potential participants will be pre-screened for major inclusion and exclusion criteria using a combination of an online survey and telephone interview. Biopsy-proven IBD will be verified in each participant’s medical record. If eligible, participants will then complete visit 1 (eligibility visit) which includes obtaining written consent and collection of self-report measures related to clinical history, demographics, and health information to determine further eligibility. Medications will be extracted from the medical record and reviewed with each participant for accuracy. Height and weight will also be collected along with vision tests for colorblindness and visual acuity with corrective lens in place. Lastly, participants will be breathalyzed and complete a urine drug screen (see schedule of assessments in Table 3).

**Table 3:** Schedule of measure administration

	Screening		Study visits					
Measures	Survey, phone interview	Visit 1	Visit 2	Visit 3	Visit 4	Visit 5	Visit 6	Visit 7
Screening and baseline measures								
Written consent, eligibility confirmation		X						
Demographics	X	X						
Vision screening		X						
Height and weight		X						
Breathalyzer		X	X	X	X	X	X	X
Urine drug test		X						
Medical history, medications	X	X						
Restless legs (CHQ, sIRLS)			X					
***Clinician-administered measures***								
HBI for CD				X				X
SCCAI for UC				X				X
***Self-report measures***								
SIBDQ (primary outcome)	X			X				X
PHQ-9				X				X
PROMIS Sleep Disturbance 8b				X				X
Morningness-Eveningness Questionnaire				X				X
Daily logs (sleep & events)			X	X	X	X	X	X
Treatment Expectation				X				
Treatment Satisfaction								X
***Objective measures***								
Wrist actigraphy			X	X	X	X	X	X
Fecal calprotectin				X				X
***Safety measures***								
BDI			X	X	X	X	X	X
C-SSRS (past 6 months)		X						
C-SSRS (since last visit)			X	X	X	X	X	X
SAFTEE					X	X	X	X

Eligible participants will be enrolled in the study at visit 2, during which some questionnaires to characterize the sample will be completed (Table 3). Participants will be given a Fitbit Charge 5™ to wear on their nondominant wrist to track their sleep and activity throughout the study protocol, along with daily logs to report their subjective experience of sleep and alcohol, drug, and medication use. One week later, at visit 3, pre-treatment outcome assessments will be administered, and participants will be randomized to the morning light treatment or TAU group (see below for more details). Treatment expectations will also be assessed at visit 3. Morning light treatment (or TAU) will then begin the morning after visit 3. Thereafter, there will be weekly visits (visits 4, 5, 6) to review each participant’s adherence to instructions (sleep timing and use of the Re-timer® in the morning light treatment group) and to systematically assess side effects (both groups). At visit 7, post-treatment outcome assessments will be administered, and study satisfaction will be assessed. Participants will be required to abstain from alcohol use within 24 h of visits, and to ensure compliance participants will be breathalyzed at the beginning of each visit.

### Randomization and blinding

Participants will be randomized at visit 3 to either 4 weeks of morning light treatment or 4 weeks of TAU in a 1:1 ratio using a minimization approach to reduce imbalances in important covariates including SIBDQ score (≤ 45 vs. > 45 to reflect moderately and severely impaired IBD–related quality of life), age 18–45 years vs. > 45 years, and sex. We will also stratify randomization by CD vs. UC and aim to enroll equal numbers of CD vs. UC and male vs. female participants. Study research assistants assessing outcomes and the clinicians assessing disease activity at study visits 3 and 7 will be blinded to treatment assignment and will be instructed not to discuss any aspect of the treatment with participants. These blinded staff will also wear badges instructing participants to not discuss the study treatment with them. Otherwise, unblinded study staff will meet with participants weekly to download data from the wrist monitor and Re-timer® device (in the light treatment group), provide feedback on adherence, and assess treatment side effects.

### Intervention: morning light treatment

The morning light treatment will be self-administered at home using the commercially available wearable Re-timer® light therapy glasses which emit a green light and is designed to optimize therapeutic wavelength (~ 500 nm, 230 µW/m^2^, 500 lx) by being close to the peak sensitivity of the circadian photoreceptors (~ 480 nm) [32, 33]. The Re-timer® device can be worn over glasses and does not interfere with ambulation, vision, reading, or computer work. Participants are instructed to start the light treatment the morning after visit 3, immediately following their assigned wake time. Their assigned wake time was their average final wake time as determined during the baseline week, or up to one hour earlier than their average wake time, to allow time for light treatment [34]. If the participant’s assigned wake time is advanced from their average wake time, then their bedtime is also shifted earlier to avoid sleep deprivation. The earliest start time for light treatment is 6am. The Re-timer® device automatically turns off after 1 h. Participants complete the 1 h light treatment sessions for 28 consecutive days at the same time each day. Participants are also instructed not to sleep or meditate (closing eyes) within four hours after their assigned light treatment start time, to avoid creating a dark pulse that can counteract the effect of the light.

Adherence is assessed via light and actigraphy data measured by a monitor (30 s epochs, Actiwatch Spectrum Plus, Respironics, Bend, OR) attached to the Re-timer®, which is reviewed with participants at the weekly study visits by unblinded staff. Research staff will contact all participants via phone call or text 10 min after their assigned light treatment start time each morning to ensure that they have started the light treatment. Participants are also given an alarm clock set to their assigned wake time to promote adherence.

### Comparator: treatment as usual

Participants assigned to TAU will be instructed at visit 3 to follow their usual ad lib sleep schedule, as they did during the baseline week. Research staff will contact these participants via phone call or text each day to encourage them to complete their daily logs. This contact, together with the weekly study visits, will ensure equivalent study contact and attention between the morning light treatment group and the TAU group.

### Outcome measures

Study outcomes will be assessed in both groups at pre-treatment (visit 3) and post-treatment (visit 7). The study assessment schedule is shown **in** Table 3.

### Self-reported outcomes

The primary outcome measure is IBD-related quality of life and this will be assessed using the Short IBD Questionnaire (SIBDQ) [35]. The SIBDQ consists of 10 items and measures the impact of IBD on social, emotional, systemic, and bowel well-being. The SIBDQ score ranges from 10 to 70 with higher scores representing better IBD-related quality of life. The SIBDQ is reliable and responsive to clinically meaningful change [36, 37]. Secondary outcome measures include the Patient Health Questionnaire-9 (PHQ-9), which is a reliable measure of the severity of depressive symptoms [38]. The PHQ-9 consists of 9 items and higher scores reflect higher levels of depressive symptoms. Sleep quality will be assessed using the PROMIS Sleep Disturbance Short-Form 8b, which is a reliable measure of sleep quality [39]. Higher scores on the PROMIS Sleep Disturbance Short-Form 8b reflect worse sleep quality.

### Clinician-rated outcomes

Clinician-rated disease activity will be assessed with the Harvey Bradshaw Index (HBI) for participants with Crohn’s disease or the Simple Clinical Colitis Activity Index (SCCAI) for participants with ulcerative colitis or IBD-unclassified [40, 41]. The HBI addresses general well-being, abdominal pain, liquidity of stool, presence of an abdominal mass, and complications, and must be completed in-person to conduct an abdominal examination. The SCCAI addresses bowel frequency, defecation urgency, blood in stool, general well-being, and extracolonic disease characteristics and can be completed in-person or remotely via video communication services.

### Exploratory outcome

We will assess for gastrointestinal inflammation by measuring fecal calprotectin. Fecal calprotectin is an inflammatory biomarker found in stool that is produced by neutrophils that infiltrate the colon and is more sensitive and specific for intestinal inflammation than systemic biomarkers derived from blood [42, 43]. Research participants will collect 1–5 gram stool samples at home the day before visit 3 (pre-treatment) and visit 7 (post-treatment). and deliver them to the study team during the study visits. The University of Michigan Central Labs will assay the samples using the commercially available INova QUANTA Flash Calprotectin Chemiluminescent Immunoassay. Participants will also complete Crohn’s disease and ulcerative colitis specific patient reported outcomes (CD-PRO and UC-PRO) that assess bowel symptoms, abdominal symptoms, systemic symptoms, daily life impact, emotional impact, and use of coping strategies [44, 45]. The CD-PRO and UC-PRO were developed in accordance with U.S. Food and Drug Administration Guidelines for use in clinical trials.

### Additional measures

At visit 2, participants will complete the Cambridge-Hopkins questionnaire [46] to screen for restless leg syndrome (RLS) which is present in 20–30% of patients with IBD [29–31]. For those that screen positive, the severity of RLS will be determined with the validated patient-report International Restless Leg Syndrome study group severity rating scale [47]. This scale assesses symptoms over the past 7 days and the total score ranges from 0 (no symptoms) to 40 (very severe symptoms). Patients who do not screen positive will be assigned a severity score of 0. The severity score will be used as a moderator in the statistical analysis (see below).

Participants will also complete several questionnaires at pre-treatment (visit 3) and post-treatment (visit 7). The Morningness-Eveningness Questionnaire (MEQ) [48] will be used as a proxy marker of circadian timing. The MEQ score correlates well with the gold standard circadian phase marker, the dim light melatonin onset, and significantly increases towards more morningness after 13–28 days of a morning light treatment [49]. This will allow us to confirm an expected phase advance or earlier shift in circadian timing in response to morning light treatment. Fatigue will be assessed with the PROMIS Fatigue Scale, where higher scores reflect more fatigue [50, 51]. Anxiety will be assessed with the PROMIS Anxiety Scale to measure anxiety, where higher scores reflect more anxiety [52, 53]. Finally, participants will also complete a brief questionnaire about their study expectations at visit 3 after they are informed of the condition they have been randomized to, and a study satisfaction questionnaire at visit 7.

Participants will wear the Fitbit Charge 5™ on their non-dominant wrist to monitor activity and sleep. The Fitbit Charge 5™ uses accelerometry and cardiac autonomic signals to estimate sleep and has been compared to gold-standard polysomnography, demonstrating differentiation of sleep and wake states superior to US Food and Drug Administration cleared actigraphy [54]. We will export data from the Fitbit Charge 5™ in 30 s intervals using Fitabase software. To augment the sleep data collected by the Fitbit device, sleep diaries will be completed, and participants will text a research email account to identify when they are trying to fall asleep (bedtime) and at their final wake time. Bedtime and rise time data through the time stamped text messages will allow for confirmation of correct sleep onset and offset and calculation of time in bed (TIB), total sleep time (TST), wake after sleep onset (WASO), and sleep efficiency (SE). These objective sleep parameters will be primarily used to verify participant adherence to the morning light treatment but will also be examined for group differences. Participants will also track their daily use of alcohol, caffeine, nicotine, medications, exercise, psychotherapy, and food timing on a daily event log.

### Safety measures

At each weekly visit after the start of treatment (visits 4–7), participants will complete a self-reported measure of physical and emotional symptoms they have experienced in the past week using the Systematic Assessment for Treatment Emergent Events (SAFTEE) as used in a previous light treatment study [55]. Unblinded research staff assess the severity of symptoms and their relevance to the study and assigned group, to ensure no significant negative effects are associated with the study participation. If severity meets a pre-determined threshold based on SAFTEE, the unblinded research staff alerts an unblinded study physician to further assess the participant’s safety and risk for an adverse event. In addition, at visits 4–7, suicidality will be assessed using the Columbia Suicide Severity Rating Scale (C-SSRS) [56], and mood worsening will examined with the Beck Depression Inventory [57]. 

### Statistical power and analysis

We plan to enroll 68 individuals with a biopsy-proven diagnosis of IBD, aged 18 years or older, who meet criteria for impaired IBD-related quality of life and report recent abdominal pain or bowel symptoms, with the aim of a final sample of 50 study participants, assuming 25% attrition during the 5-week study period. A 9-point change in SIBDQ score is considered clinically meaningful and corresponds to a standardized effect size of 1.0 SD [35, 58, 59]. This proposed sample of 50 participants (25 participants in the morning light treatment group and 25 participants in the TAU group) provides ≥ 80% power to detect a clinically meaningful change in our primary outcome measure (SIBDQ) with a two-tailed alpha of 0.05.

We will conduct an intention-to-treat and completer analyses. We will report descriptive statistics of the data by each assessment time (pre-treatment, post-treatment) using appropriate summary statistics. We will analyze both primary and secondary outcomes using a mixed-effects longitudinal data model with participants as random intercepts to account for between participant variability. Predictors will include a post-randomization time indicator, intervention group indicator, and the interaction of time by group. We will examine the extent and pattern of key missing outcomes data and if missingness is greater than 15%, we will assess for baseline characteristics predictive of missingness post-randomization and will include those baseline characteristics as covariates in the final model. While clinician-rated disease activity will be examined as a continuous variable, we will also examine the proportion of participants who show clinical response and/or clinical remission as a binary variable using a generalized linear mixed model with logit link. Clinical response is defined as a decrease in SCCAI score ≥ 3 or a decrease in HBI score ≥ 3. Clinical remission is defined as an SCCAI score of ≤ 2 or an HBI score of < 5.^40,41^

We will explore steroid use, severity of RLS, and biological sex as potential moderators of any treatment effects. We will also perform subgroup analyses to explore differences in responses to morning light treatment between participants with CD versus UC/IBD-unclassified to inform future studies.

## Discussion

This protocol manuscript describes a single center randomized controlled trial of morning light treatment vs. TAU in IBD. This study will provide insight into the effect of morning light treatment on IBD-related quality of life, depression, sleep quality, and clinical disease activity for patients with IBD to inform its role as an adjunctive therapy for patients with active symptoms. Despite the availability of effective IBD-targeted pharmacological therapies, many patients with IBD continue to suffer from inadequate disease control and impaired health-related quality of life. Many patients also identify preferences for non-pharmacological treatment options, but few evidence-based non-pharmacologic strategies exist for IBD. Therefore, morning light treatment has the potential to play an important role in IBD treatment if found to be efficacious. Based on published literature and our prior work in chronic pain populations, morning light treatment has the potential to improve not only mood and sleep in IBD, but also improve gastrointestinal symptoms and possibly even intestinal inflammation. Even if morning light treatment were to only improve mood and sleep in patients with IBD, this treatment would assist at least 50% of IBD patients who suffer from such symptoms [4–8]. 

The strength of this study includes the randomized trial design and control group, blinding of study staff directly involved in assessing study outcomes, and a well-described conceptual model of the potential mechanistic relationship between morning light treatment and study outcomes. The anticipated study limitations include exclusion of patients with an ileostomy, colostomy, ileoanal pouch, ileorectal anastomosis, or short bowel syndroe which will limit generalizability of study findings to such patients who have undergone these major surgical procedures. Further, the study period is limited to five weeks and so long-term effects of morning light treatment in IBD will not be evaluated. However, we will plan to test the long-term effects of morning light treatment in IBD in future work.

This is the first NIH-funded randomized controlled trial of morning light treatment in IBD and has the potential to improve our understanding of the impact of morning light treatment on patients with IBD and fill a gap in adjunctive non-pharmacological IBD treatment. This study will also increase our understanding of the mechanistic relationships between sleep, mood, clinical disease activity, and IBD symptoms. If found to be efficacious, morning light treatment can be further examined in a larger clinical trial with the possibility of remote study visits to increase ease of participation to patients and a longer follow up period.

## Data Availability

No datasets were generated or analysed during the current study.

## Abbreviations

IBD: Inflammatory bowel disease
TAU: Treatment as usual
CD: Crohn’s disease
UC: Ulcerative colitis
PHQ-9: Patient Health Questionnaire
HBI: Harvey Bradshaw Index
SCCAI: Simple Clinical Colitis Activity Index
SIBDQ: Short Inflammatory Bowel Disease Questionnaire
CD-PRO: Crohn’s disease-patient reported outcomes
UC-PRO: Ulcerative colitis-patient reported outcomes
RLS: Restless leg syndrome
TIB: Time in bed
TST: Total sleep time
WASO: Wake after sleep onset
SE: Sleep efficiency
SAFTEE: Systematic Assessment for Treatment Emergent Events
